# Do subtle cultural differences sculpt face pareidolia?

**DOI:** 10.1038/s41537-023-00355-y

**Published:** 2023-05-04

**Authors:** Valentina Romagnano, Alexander N. Sokolov, Andreas J. Fallgatter, Marina A. Pavlova

**Affiliations:** Department of Psychiatry and Psychotherapy, Tübingen Center for Mental Health (TüCMH), Medical School and University Hospital, Eberhard Karls University of Tübingen, Tübingen, Germany

**Keywords:** Neuroscience, Schizophrenia

## Abstract

Face tuning to non-face images such as shadows or grilled toasts is termed *face pareidolia*. Face-pareidolia images represent a valuable tool for investigation of social cognition in mental disorders. Here we examined (i) whether, and, if so, how face pareidolia is affected by subtle cultural differences; and (ii) whether this impact is modulated by gender. With this purpose in mind, females and males from Northern Italy were administered a set of Face-n-Thing images, photographs of objects such as houses or waves to a varying degree resembling a face. Participants were presented with pareidolia images with canonical upright orientation and display inversion that heavily affects face pareidolia. In a two-alternative forced-choice paradigm, beholders had to indicate whether each image resembled a face. The outcome was compared with the findings obtained in the Southwest of Germany. With upright orientation, neither cultural background nor gender affected face pareidolia. As expected, display inversion generally mired face pareidolia. Yet, while display inversion led to a drastic reduction of face impression in German males as compared to females, in Italians, no gender differences were found. In a nutshell, subtle cultural differences do not sculpt face pareidolia, but instead affect face impression in a gender-specific way under unusual viewing conditions. Clarification of the origins of these effects requires tailored brain imaging work. Implications for transcultural psychiatry, in particular, for schizophrenia research, are highlighted and discussed.

## Introduction

Face pareidolia [from the ancient Greek παρά (para) – ‘next to it’ and είδωλον (eidolon) – ‘shape, sign, image’, a kind of *apophenia*, a tendency for perceiving meaningful connections between unrelated elements] reflects high tuning to a coarse face scheme (such as *two eyes above a mouth*) available in face-like non-face images such as waves, shadows or grilled toasts^1,2^. In recent years, face pareidolia has elicited great research interest^3–25^, primarily, because non-face face-like images do not contain any single element that fosters face processing. The other benefit (that is of special value in clinical settings, in particular, in psychiatry) is using unfamiliar images^26^. Furthermore, non-face stimuli help to eliminate possible own-culture biases^27^ that persist in cross-cultural studies even with face inversion^28^.

Cross-disease studies in individuals with mental and neurodevelopmental disorders such as schizophrenia (SZ)^29,30^, autism spectrum disorders (ASD)^6^, Down syndrome^7^, Williams syndrome^4^, or in adolescents born prematurely^10^ reveal substantial, albeit specific for each single condition, deficits in face tuning. These deficits are characterized by a rather dissimilar disease-specific dynamics^10,29^.

Gender (a social construct referring to social roles, norms, gender identification, etc.) of beholders is an important variable to consider in face perception research^9,11^. However, only a handful of studies are available on gender differences in face pareidolia, and the outcome is rather inconclusive. On a spontaneous recognition task with Face-n-Food Arcimboldo-like images (consisting of compositions of fruits and vegetables) presented in ascending order from the least to most face resembling, females are found to be more face-sensitive than their male peers^3^. However, no gender differences occur on the same task in patients with major depressive disorder (MDD)^31^. The lack of gender differences is reported when rating perceived gender, age, and emotional expression of face-like non-faces^32^. Yet women attribute greater face-likeness to non-face images such as clocks or backpacks than men^11^. Moreover, face pareidolia is positively tied with face likability in a gender-specific way, occurring only in female perceivers^5^.

Culture is another vital factor that sculpts cognitive skills. For example, differences were found between Western and Asian populations in working memory, attention, and emotion recognition^33–36^. A large body of research focuses on rather pronounced cultural differences, for instance, between European or North American and East Asian or African individuals, who contrast in their philosophy of life and the global world perception. In general, Westerners tend to analytically focus on salient objects, while Asians engage more holistic processes^37,38^. In harmony with this, people with different cultural background differ in cues they use for face processing: East Asians prioritize global information^39–44^ fixating more on the center of faces (a nose area) and less on the eyes and mouth areas than Westerners^39,43^. On the same wavelength, when scanning dynamic faces, differences in eye movements are reported between British/Irish and Japanese persons, with noticeable mouth scanning in British/Irish individuals and Japanese individuals engaging in greater eye and central face looking^45,46^. Dissimilarities are observed already at early stages of face processing, with East Asians relying more on coarse-grained rather than fine-grained information than Westerners^47^. The outcome suggests that marked differences occur in the very nature of face information extracted by individuals with different cultural/ethnical background.

Yet more subtle differences may also affect social cognition. For instance, Italian children aged 3- to 15- years are more skillful in affect recognition than their peers from Finland and the United States^48^. German and Italian adolescents are reported to differ on the Reading the Mind in the Eyes Test (RMET), with higher scores in German as compared to Italian men^49^. Previous research with non-face face-like images found that even subtle cultural differences profoundly modulate gender impact on face tuning: males from the French-speaking part of Switzerland exhibited higher sensitivity to faces than their peers from the Southwest of Germany, whereas no difference in face tuning occurred between females^8^.

In view of the current world globalization with an enormously increasing number of immigrants, in particular, those maintaining their original customs and traditions, cross-cultural investigation of social cognition is of immense value for understanding of mental diseases and psychiatric symptoms. This implies that the same health care institution may face different clinical manifestations of the same mental illness (that potentially affects making diagnoses) as well as requirements for different efficient treatment strategies (such as psycho- and occupational therapy) of individuals of different ethnicity.

The present work intended to clarify: (i) whether subtle cultural differences shape face pareidolia; and (ii) whether, and, if so, how, gender differences in face pareidolia are modulated by cultural background. To this end, young adults from Northern Italy (Milan area) were administered a set of face-like Face-n-Thing images with canonical upright and inverted to 180° in the image plane orientation. Display inversion is used as a control condition in studies with face-like non-faces^50–52^, since (as shown by our recent research^9,30,53^) it substantially lessens face resemblance. Inversion provides a proper control for face pareidolia, because an overall amount of intra-stimulus information remains the same with both orientations.

## Methods

### Participants

In total, the data sets of 90 participants were collected. Forty-four native Italian young adults, 22 females and 22 males, aged 18–37 years were recruited and examined in the North of Italy (Milan area). One male participant turned out to be an outlier. This left 43 participants in the final sample (21 males/22 females). Females were aged 25.91 ± 4.01 years (mean ± standard deviation, SD; median, Mdn, 24 years; 95% confidence interval, CI [24.13, 27.69]), and males were aged 24.38 ± 4.06 years (Mdn, 24 years, 95% CI [22.53, 26.23]). No age differences were found between Italian males and females (Mann–Whitney test, *U* = 200.5, *p* = 0.458, two-tailed, n.s.). None of them had a history of neurological or psychiatric disorders (including ASD, SZ, or MDD that potentially may affect visual social cognition) and regular intake of medication. The data of 46 native Germans [22 females, aged 24.27 ± 2.60 years and 24 males, aged 24.46 ± 4.99 years, with no age difference between them; *t*(44) = 1.16, *p* = 0.874, two-tailed, n.s.] were collected earlier in Southwestern Germany, Tübingen^9^. As no age differences between females and males were found in both groups, Italian and German groups of participants had been compared in respect to age: Italians were aged 25.16 ± 4.06 years; Mdn, 24 years, 95% CI [23.91, 26.41], and Germans aged 24.37 ± 3.99 years; Mdn, 23.5 years, 95% CI [23.19, 25.55]; with no age differences between the groups (Mann–Whitney test, *U* = 870.0, *p* = 0.328, two-tailed, n.s.). The number of participants was determined by demands of statistical data processing. The present sample size calculation made use of the only available behavioral study on cultural/gender impact on face pareidolia^8^. The inclusion criteria were having German/Italian as mother tongue, to be born and/or grew up in the respective country from early childhood. As in our previous studies^3,5,8,9,30,31^, gender was self-identified by participants; there were also no female participants with extreme masculine appearance and behavior, and vice versa.

Participants were run individually in a face-to-face experiment. They were naïve as to the purpose of the study, and none had previous experience with such images and tasks. All observers had normal or corrected-to-normal vision. The study was conducted in line with the Declaration of Helsinki and was approved by the local Ethics Committee at the Medical School, Eberhard Karls University of Tübingen. Informed written consent was obtained from all participants. Participation was voluntary and the data were processed anonymously.

### Task and procedure

**Fig. 1:**
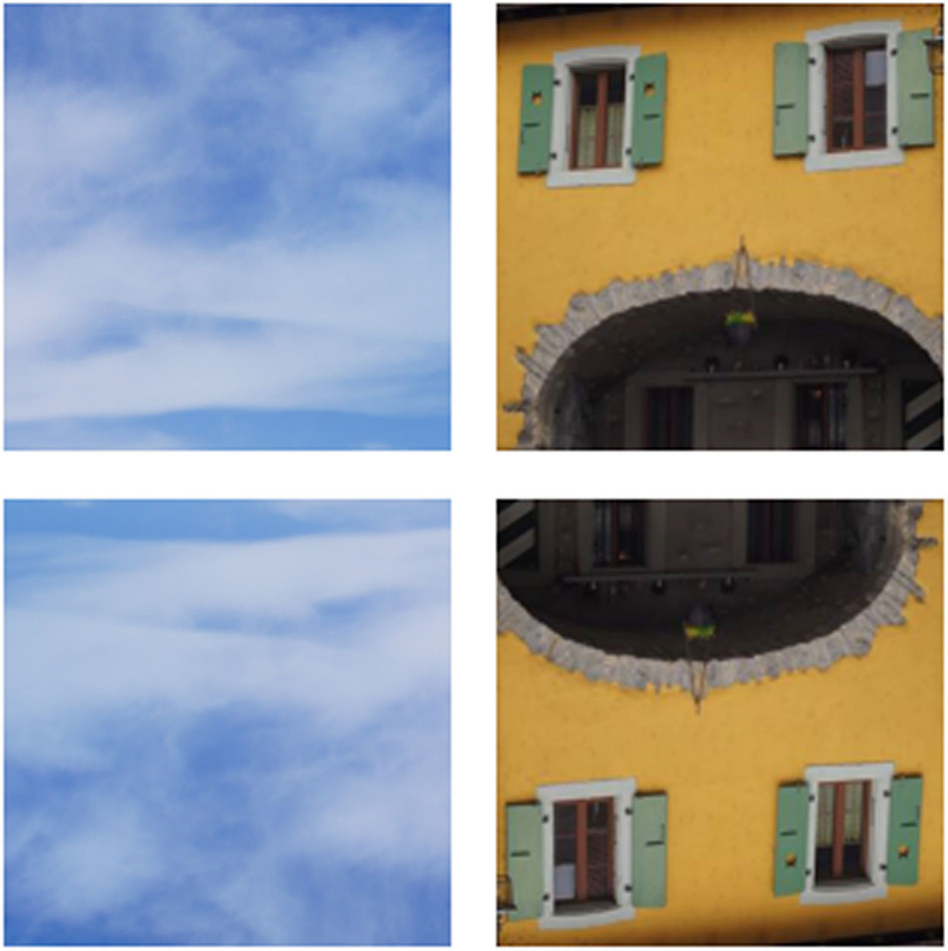
Examples of the Face-n-Thing images with canonical upright (top) and inverted (bottom) display orientation. When presented with upright orientation, the image on the left is one of the least resembling a face and the image on the right, one of the most resembling a face. Right panel is from^9^*, PLoS ONE*, Creative Commons Attribution License [CC BY].

The task and procedure are described in detail elsewhere^9,30^. In brief, participants were administered a computer version of the task by using Presentation software (Neurobehavioral Systems, Inc., Albany, CA, USA). They were presented with a set of Face-n-Thing images (such as houses and clouds; Fig. 1), in varying degree resembling a face. The stimuli subtended a visual angle of 9.8˚ × 9.8˚ at an observation distance of 70 cm. The images were presented in pseudo-randomized order, one by one for 1 s with either canonical or inverted orientation in 3 runs with a short break between the runs. In total, each experimental session consisted of 168 trials (14 images × 2 types [original/mirror image] × 2 display orientations [upright/inverted] × 3 runs). No more than three images with the same orientation (either upright or inverted) appeared consecutively; in this way, a possible adaptation of the visual system to display orientation was prevented. On each trial, in a two-alternative forced-choice (2AFC) task, participants had to indicate whether they have an impression of a face by pressing a respective key. Participants were explicitly told that there were no correct or incorrect responses on the task, and they had to rely solely upon their own visual impression. They were asked to respond as fast as possible after stimulus offset during an inter-stimulus interval (after stimulus offset and till onset of the next stimulus right after participant’s response). During this interval, a white fixation cross was displayed in the center of the screen for a maximum duration jittered from 4 to 6 s. If participants failed to respond within this period, the next trial automatically started. No immediate feedback was provided. Instructions were carefully explained to participants and their understanding had been proven with pre-testing (about 10 trials). Participants were run individually. None had previous experience with such stimuli and task. The testing procedure lasted for about 25–30 min.

### Data processing and analysis

Prior to statistical data processing, all data sets were routinely analyzed for normality of distribution by using Shapiro-Wilk tests with subsequent use of either parametric (for normally distributed data) or non-parametric statistics. For not normally distributed data sets, additionally to means and SDs, Mdns and 95% CI were reported. Inferential statistics was performed by both between-subject and mixed-model analyses of variance (ANOVAs), and post-hoc pairwise comparisons by using two-tailed false discovery rate (FDR) corrected *t*-tests with software package JMP (Version 16, SAS Institute, Cary, NC, USA). Non-parametric statistics (Mann–Whitney test and Wilcoxon signed-rank test) was computed for between- and within-group comparisons, respectively, with MATLAB (version 2022a, The MathWorks Inc., Natick, MA, USA^54^).

## Results

### Face response rate

**Fig. 2:**
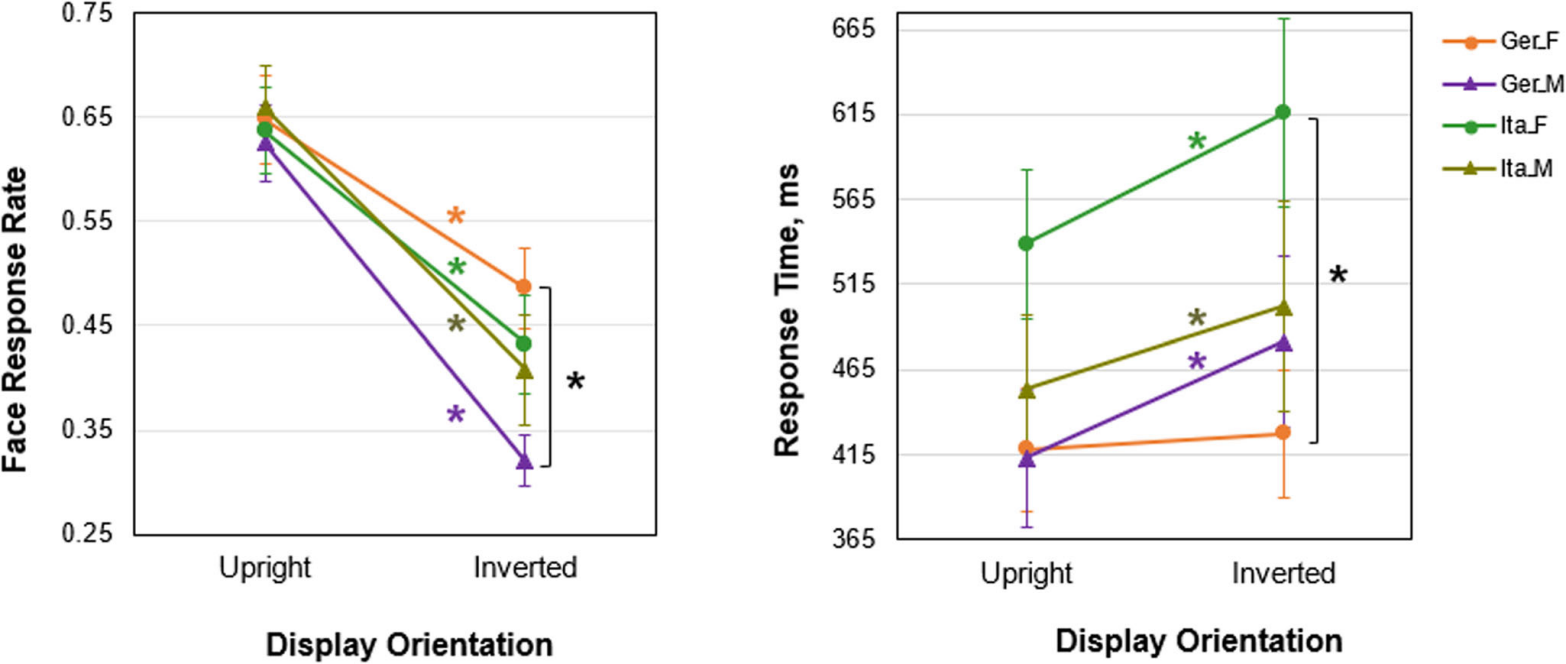
Effects of cultural background on face pareidolia with upright and inverted display orientations. Left panel represents mean face response rate with upright orientation and inversion in German and Italian females (orange and green blobs, respectively) and in German and Italian males (violet and olive triangles, respectively). Right panel represents mean face response time with upright and inverted orientations in German and Italian females (orange and green blobs, respectively), and German and Italian males (violet and olive triangles, respectively). Vertical bars represent ± SEM. Asterisks indicate significant differences: orange – display orientation effect in German females, green – in Italian females, violet – in German males, and olive – in Italian males, and black – differences in face response rate between German females and males (left) and in RT between German and Italian females in inverted orientation (right; *p* < 0.05). The data in Germans were reported earlier^9^.

Individual face response rates were submitted to a three-way mixed-model ANOVA with a within-subject factor Display Orientation (upright/inverted) and between-subject factors Gender (female/male) and Culture (Italian/German). A main effect of Orientation was significant (*F*(1,85) = 63.8, *p* < 0.001; effect size, partial eta-squared *η*^2^ = 0.273) with higher face pareidolia rates with upright display orientation than with display inversion (Fig. 2). Neither main effects of Culture (*F*(1,85) = 0.24, *p* = 0.625, n.s.) nor of Gender were significant (*F*(1,85) = 2.74, *p* = 0.10. n.s.). Interactions of Culture by Gender (*F*(1,85) = 2.62, *p* = 0.108, n.s.), Culture by Orientation (*F*(1,85) = 0.01, *p* = 0.939, n.s.), and Gender by Orientation (*F*(1,85) = 2.71, *p* = 0.102, n.s.) were not significant. Significant effects of inversion on face pareidolia in German females and males were reported earlier^9^. Also in Italians, face pareidolia was substantially impaired by display inversion (for females, 0.64 ± 0.19 with upright orientation; 0.43 ± 0.22, with inversion; *t*(21) = 3.53, *p* < 0.001, FDR corrected for multiplicity throughout; Cohen´s *d* = 0.797; for males, 0.66 ± 0.18, with upright orientation; 0.41 ± 0.24, with inversion; *t*(20) = 4.25, *p* < 0.001, Cohen´s *d* = 0.862).

As inversion substantially affects face pareidolia, we dismantled the impact of Gender and Culture with upright and inverted orientations, separately, in a two-way ANOVA with two between-subject factors Gender (female/male) and Culture (Italian/German). With upright orientation, neither main effects of Gender (*F*(1,85) = 0.0001, *p* = 0.993, n.s.) and Culture (*F*(1,85) = 0.09, *p* = 0.766, n.s.) nor interaction between these factors (*F*(1,85) = 0.33, *p* = 0.568, n.s.) were significant.

By contrast, with inversion, a main effect of Gender was significant (*F*(1,85) = 5.21, *p* = 0.025; effect size, eta-squared *η*^*2*^ = 0.557), with a greater face response rates in females as compared to males. With inversion, a main effect of Culture was not significant (*F*(1,85) = 0.15, *p* = 0.697; n.s.), whereas a Culture by Gender interaction had a weak tendency to reach significance (*F*(1,85) = 2.85, *p* = 0.095). As reported earlier^9^, with inversion, German participants exhibited gender differences in face response rate. The inversion effect was more pronounced in males: In females, display inversion resulted in a drop of face impression from the Face-n-Thing images by 25%, whereas in males face impression fell down by about 50%. By contrast, no gender difference in face response rate with inversion was found in Italians (*t*(41) = 0.41, *p* = 0.681, n.s.; FDR corrected for multiplicity throughout). Neither German and Italian females (*t*(42) = 0.91, *p* = 0.485, n.s.) nor German and Italian males significantly differed in face tuning with display inversion (*t*(44) = 1.48, *p* = 0.287, n.s.).

### Face response time

With display inversion, one Italian male participant did not experience face pareidolia at all, and, therefore, the data sets of 20 male Italians entered the response-time (RT) data analysis. In general, a RT analysis has only a secondary role, since participants had been asked to respond as soon as possible after the stimulus offset. Individual data on RT (for trials, on which images elicited face impression) were submitted to a three-way mixed-model ANOVA with a within-subject factor Display Orientation (upright/inverted) and between-subject factors Gender (female/male) and Culture (Italian/German). A main effect of Culture was significant (*F*(1,84) = 9.23, *p* < 0.003; effect size, partial eta-squared *η*^*2*^ = 0.052) with shorter RT for Germans as compared to Italians (Fig. 2). A main effect of Orientation had a weak trend to reach significance (*F*(1,84) = 2.91, *p* = 0.089), and a main effect of Gender was not significant (*F*(1,84) = 0.86, *p* = 0.356, n.s.). A Culture by Gender interaction showed a weak trend to reach significance (*F*(1,84) = 2.78, *p* = 0.098), while interactions Culture by Orientation (*F*(1,84) = 0.28, *p* = 0.595, n.s.) and Gender by Orientation (*F*(1,84) = 0.15, *p* = 0.697, n.s.) were not significant.

As reported earlier^9^, German males were slower in response to images eliciting face impression with inversion as compared with upright orientation, whereas no difference in RT between upright and inverted orientations occurred in females. In Italians, RT was longer with display inversion (for females, 538.755 ± 204.464 ms, with upright orientation; and 615.891 ± 259.253 ms, with inversion; *t*(21) = 2.65, *p* = 0.032, Cohen’s *d* = 0.35, FDR corrected for multiplicity throughout; for males, 456.998 ± 203.215 ms, with upright orientation; and 527.139 ± 264.568 ms, with inversion; *t*(19) = 2.30, *p* = 0.036, Cohen’s *d* = 0.218; Fig. 2).

Similar to the face response rate analysis above, we performed two-way ANOVAs with factors Culture and Gender on RT with upright and inverted orientations, separately. With upright orientation, a main effect of Culture approached significance (*F*(1,84) = 3.93, *p* = 0.051; Germans tended to respond faster than Italians), whereas a main effect of Gender (*F*(1,84) = 1.08, *p* = 0.302, n.s.) as well as an interaction of these factors (*F*(1,84) = 0.86, *p* = 0.358, n.s.) were not significant. With upright orientation, like in Germans^9^, RT didn’t differ between Italian males and females (*t*(40) = 1.36, *p* = 0.356, n.s.; Fig. 2). Difference in RT between Italian and German females did not survive corrections for multiple comparisons (*t*(42) = 2.06, *p* = 0.170, n.s.).

With inversion, a main effect of Culture was significant (*F*(1,84) = 5.32, *p* = 0.024, eta-squared *η*^*2*^ = 0.058; with shorter RT in Germans), whereas a main effect of Gender (*F*(1,84) = 0.120, *p* = 0.730, n.s.) as well as Culture by Gender interaction (*F*(1,84) = 1.95, *p* = 0.167, n.s.) turned out to be not significant. As with upright orientation, and again like in Germans^9^, with inversion no gender difference in RT was found in Italians (*t*(40) = 1.21, p = 0.463, n.s.). With inversion, German females were faster than Italian females (*t*(42) = 2.62, *p* = 0.042; Cohen’s *d* = 0.79), whereas German and Italian males did not differ in RT (*t*(43) = 0.64, *p* = 0.523, n.s.).

## Discussion

In the present study, we explored a potential impact of cultural background on face pareidolia, the ability to seeing faces in non-face images. With this purpose in mind, healthy Italian females and males were administered a set of the Face-n-Thing images (photographs of waves, houses, clouds, etc.) with canonical and inverted display orientations. The findings were compared with the data sets collected in the Southwest of Germany. The main outcome (Fig. 2) indicates: (i) with upright orientation, neither cultural background nor gender affects face pareidolia; and (ii) as expected, display inversion generally hampers face pareidolia irrespective of cultural background and gender of observers. However, while inversion leads to a drastic reduction of face impression in German males as compared to females, no gender differences occur in Italians.

### Cultural impact on face pareidolia

As to our knowledge, the present study for a first time indicates the absence of subtle cultural differences in face pareidolia: with canonical display orientation, no differences were found between Italians and Germans. Earlier, males from the French-speaking part of Switzerland were reported to exhibit higher face tuning to Arcimboldo-like Face-n-Food images than their peers from Southwestern Germany, though Swiss and German females did not demonstrate any differences^8^. Several methodological reasons may account for this discrepancy: (i) *Different types of images*. The Face-n-Thing images were used here instead of Face-n-Food images earlier. Indeed, diverse mechanisms may underlie face pareidolia elicited by the Face-n-Thing images representing photographs of natural objects and scenes and the Face-n-Food images that were created of food ingredients on purpose to resemble a face. (ii) *Different experimental design*. A 2AFC paradigm was used here instead of a spontaneous recognition/open-end task earlier. The latter paradigm is obviously less restrictive, leaving more space for individual and cultural differences. (iii) *Different presentation mode*. Each Face-n-Thing image in this study was presented to observers several times in a pseudo-randomized order, whereas in the Face-n-Food paradigm, the images were presented only once in a fixed, predetermined order from the least to most face resembling. The latter procedure appears to be more sensitive to cultural, gender, and group differences at large. Finally, one can assume that due to some historical reasons, cultural differences between Southwestern Germans and Northern Italians may be less pronounced than between Southwestern German and French-speaking Swiss populations. Yet, a few studies report the impact of even subtle cultural differences between Western populations and Italians on social cognition (affect recognition^48^ and reading language of the eyes^49^).

With canonical orientation, in both Italian and German samples, no gender differences in face pareidolia were found. Only a very few previous studies were aimed at clarification of this issue, and the outcome is rather controversial. By using upright face-like non-faces, the VPP (vertex positive potential), a component of the event-related potential (ERP), was found to be larger for faces than for face-like non-faces in males, whereas in females, it was equal for faces and face-like non-faces. No sex difference (a neurobiological construct) occurred at early processing stages, N170 level^11^. Behavioral gender differences were not reported, since the task used (to respond to images of animals presented along with faces and face-like non-faces) did not afford such analysis. Yet women attributed higher face-likeness scores to non-faces than men^11^. On a spontaneous/open-end recognition task with Arcimboldo-like Face-n-Food images presented in ascending order, females exhibited lower thresholds for face pareidolia^3^. Yet, no gender differences occurred on the same task in patients with MDD^31^. Face pareidolia was shown to be tied with gender-specific impressions: although images most resembling a face elicited more female-face responses in both female and male observers, in females only, face pareidolia was positively linked to face likability^5^. No gender differences were found when rating perceived gender, age, and emotional expression of face-like non-face images^32^. Keeping in mind that developmental and animal studies suggest the sensitivity to a coarse face scheme either emerges early in the lifespan or exists as an innate predisposition^55^, a kind of face detection template may be sex-independently hardwired in the brain. Impact of gender (as a social construct reflecting social roles, norms, stereotypes, practices as well as gender identification) on face pareidolia may be rather of secondary origin, with substantial fluctuations over the lifespan, and, therefore, rather variable in the modern Western society.

### Face inversion effect

For a long time, it has been well recognized that display inversion impairs processing of real faces and facial affect recognition leading to a lessening in accuracy of face recognition by about 15–25%^56–58^. Although display inversion was used as a control condition in a few (brain imaging) studies with face-like non-faces^50–52^, only recently it had been rigorously shown that, and to what extent, it affects face pareidolia^9,30^. The present study yields a further support for the face inversion effect (FIE) in face-pareidolia images: irrespective of cultural background, inversion severely hampers face resemblance. Indeed, in the absence of clear face-impression triggering cues (such as a nose), with inversion, a face scheme does not work properly and, therefore, some additional efforts (for instance, image normalization) may be required for gaining face impressions^9^.

The most striking outcome of the present work is that even subtle cultural differences sculpt the FIE in face-pareidolia images. While inversion led to a drastic reduction of face impression in German males as compared to females (in females, inversion resulted in a drop of face impression by 25%, whereas in males by about 50%)^9^, in Italians, no significant gender differences were found: in females, face pareidolia with inversion was diminished by 33% and in males, by 38%. As discussed earlier^9^, it is thought that: (i) in general, females possess rather holistic perceptual style (whereas males rather local one)^3,5,8,9,59^, and (ii) display inversion heavily affects holistic perception. In accord with this view, display inversion should more severely affect females that was not the case. The other possibility is that with display inversion, females and males may use different (either parallel or serial) perceptual strategies. When processing face-pareidolia images with display inversion, less efficient serial strategy may result in greater difficulties in German males. In addition, with both canonical and inverted orientations, German males may more strictly follow instructions to respond when face impression does arise spontaneously, whereas females (intentionally or not) try to normalize inverted images. By contrast, Italian females and males (similar to French-speaking Swiss individuals^8^), may exhibit less pronounced gender differences in perceptual style and/or strategies (that reflect socio-cultural impact on upbringing and socially desirable gender-specific behavioral styles). Accordingly, this may result in the lack of gender differences in face pareidolia with display inversion. In a nutshell, subtle cultural differences do not shape face pareidolia, but under unusual viewing conditions (such as display inversion), affect face impression in a gender-specific way.

### Implications for psychiatry and future directions

Efficient reading of body and face language is a key component of social competence^60–73^. This ability is reported to be compromised in most mental disorders including SZ and ASD^74–79^. As face tuning in face-pareidolia images occurs without being explicitly fostered by familiar face cues, these stimuli represent a valuable tool for investigation of face processing, in particular, when studying clinical populations^3–10,29–31,53^.

First of all, there is little doubt that psychiatry does not exist outside of socio-cultural context: it is not contextless, as well as it is not mindless and brainless. Recent work delivers evidence for fluid cultural shaping of mental disorders, in particular, their experience and expression: clinical manifestation as well as severity and origins of symptoms may vary substantially depending on the cultural background^80–83^. For example, while standard (Western) diagnostic tools for MDD focus on mood, lack of motivation, and chronic fatigue, Chinese individuals with depression often complain of stomach pain and headache^84^. Changes in appetite and in weight load on different factors in Spanish speakers from Latin America, English-speaking Western countries, and English speakers from Southeastern Asia, but not in Chinese and Russian individuals, whereas core depression symptoms tend to load with physical symptoms in most ethnic samples except Russians^85^. Substantial differences in ASD symptom severity and caregiver endorsement are reported in persons from Greece, Italy, Japan, Poland, and the United States^86^.

In SZ, cultural differences are reported in prevalence of symptoms such as visual and auditory hallucinations (e.g., between the United States and India^87^). Among patients from Austria, Lithuania, Poland, Georgia, Ghana, Nigeria, and Pakistan, visual hallucinations are more frequent in patients from the West of Africa, while auditory hallucinations are rare in Austria and Georgia^88^. While in US patients with SZ, suicide attempts correlate among others with pattern of symptoms and educational status, no such associations are found in the Indian sample^89^. Early non-psychotic deviant behavior (such as poor socialization, extreme fears/chronic sadness) is in a different way associated with disease outcome: for example, compared to US patients, African patients with early fears/chronic sadness are 3 times more likely to attempt suicide^90^.

Culture affects not only development, structure, and functioning of the typically developing brain, but cultural background is reported to be associated with the brain aberrations and abnormalities in psychiatric conditions. For instance, widespread grey matter reduction over a number of the frontal and temporal cortices (e.g., the left inferior and middle temporal gyri) in SZ differs between German and Japanese patients^91,92^, which may contribute to differences in clinical manifestation. Therefore, it is of substantial importance to tease out potential ways of cultural impact on cognition and behavior. Apparently, social cognition in mental disorders is particularly prone to cultural influences^93,94^. Cross-cultural comparison of patients and typically developing controls from Denmark and China on social cognition skills (assessed by Brüne’s Picture Sequencing and Animated Triangles tasks) revealed that the cultural/ethnical background influences both patients and controls^95^. Development of novel tools for snap-shot clinical examination of social cognition as well as improvement of already existing methods and instruments requires careful consideration of possible impact of even subtle differences in cultural background and social practices.

In a nutshell, the outcome of the present study indicates: (a) Subtle differences in cultural background between Southwestern German and Northern Italian individuals do not shape face pareidolia. Moreover, cultural impact on face pareidolia is not modulated by gender; (b) Display inversion is a valuable experimental manipulation providing for a proper control when studying face pareidolia and underlying neural networks. Irrespective of subtle cultural differences, inversion substantially diminishes face pareidolia, albeit upright and inverted images consist of the same number of elements representing the same relative spatial arrangement; and (c) Possible cultural differences in face pareidolia should be taken into account when conceiving and designing behavioral and brain imaging studies and elaborating data processing: whereas in German males, display inversion efficiently reduces face pareidolia impression, in females, this effect is much less pronounced. No gender differences occur in Italians. Clarification of the origins of these effects calls for further tailored experimental work.

## Data Availability

The data supporting the conclusions of this paper are either included in the paper or will be made available by the authors upon request to any qualified researcher.
